# Use of humour in psychiatric practice: Can we do it properly?

**DOI:** 10.4103/0019-5545.31564

**Published:** 2006

**Authors:** G. Prasad Rao

**Affiliations:** * Consultant Psychiatrist, Asha Hospital, Hyderabad Flat No. 102, Sai Vishnu Apartments, Domalguda 1-2-26, Hyderabad 500029, Andhra Pradesh; e-mail: prasad40@gmail.com

[The following are invited commentaries on the ‘Viewpoint’ by Dr G. Swaminath: ‘Jokes A Part: In defence of humour’ (Indian J Psychiatry 2006;48:177–80)—Ed.]

Dr G. Swaminath's viewpoint is to be viewed not as a ‘joke’ but taken seriously. The ‘art of psychiatry’ needs to be restated and reinforced in clinical practice.

Though without doubt his address reflects his personality, the presentation of his viewpoint comes across very strongly and he tries to tell us how ‘humour’ should be used in clinical practice, both in history taking and in some measure in psychotherapy. In 1979, Norman Cousins talked about the therapeutic influence of humour, but it is amazing that no ‘serious’ thinking has yet been done in terms of investigating the use of humour in psychiatry. Except Kahn's (1989) explanation of the primary function, there does not seem to be any work which is worth reading, either as theory or of use of humour in psychotherapeutic techniques. In a sense, Swaminath's elaborations should be viewed from a ‘critical angle’. His snapshots are just the appetizers, but a look at them gives the obvious message that most clinicians use some of the techniques in rapport-building. However, anecdotal reports do not add up to great evidence, especially in this era of evidence-based psychiatry. There is a need of converting the pearls of Swaminath to an assessable method, apart from devising methods for use of humour in psychiatry.

In psychiatric practice there is always an exchange of emotions and thoughts and the psychiatrist has to use his learnt skill in understanding the client and help him with the acquired knowledge. The interaction is quite intimate and depends on the personality of the psychiatrist. A psychiatrist should be a good communicator. What Swaminath tells us is that humour should be an integral skill of a good communicator. Humour helps in rapport-building at various stages in clinical interviews and in some psychotherapies as well.

My personal experiences of using humour are mixed. Initially, I used to bring up some relevant jokes during psychotherapeutic sessions especially when the client was using strong defence or was silent. I would employ the ongoing session content to bring out some emotional exchange which the patient was possibly trying to hide or avoid. I would use some known stories of Birbal or Tenaliramalinga, both famous for natural humour. Sometimes though, patients will not react, but more often than not most would react with a bit of cheer and I would see the session progress further. It would definitely smoothen the nerves of both of us involved in the therapy. At times humour would make new openings for the client to restart the conversation and would bring out some emotional issues. Humour would make the task easy particularly with patients with obsessive traits where there used to be some resistance in psychodynamic sessions. I use humour more in my interviews with children especially when they are unable to express themselves. I use humorous stories using their hero figure as the role model, which make them relax. Children would start the next session with their humorous story and I would try to direct them into their problems. Humour does help in dealing with children.

I use humour in marital therapies too where a careful use of humour would help in easing the tough periods during a session. Sometimes humour is used in interpretation; it helps to explain some major conflicts which affect the couples all the time and recur in the session. Lighthearted interpretation would often drive home the resolution of the conflict.

But the serious question obviously is: Can humour be taught to the budding psychiatrist? Much like psychotherapy training, can we formalize training of humour, if it is useful?

I cannot conclude, but with a wish, that young researchers should take up some of the precepts and concepts of Swaminath and conduct some experimental research. Though we know research in psychotherapy to test this hypothesis needs a difficult design, we should try to study and bring out the usefulness or otherwise of the use of humour in psycho-therapy. Likewise, I think we should try to see how humour can be used in psychiatric interviews as well. It will definitely help people like Swaminath to practice what they believe in, i.e. the ‘art of psychiatry’, with more conviction. Otherwise his effort is yet another individual ‘opinion’.
